# Patterns of preserved and impaired spatial memory in a case of developmental amnesia

**DOI:** 10.3389/fnhum.2015.00196

**Published:** 2015-05-11

**Authors:** R. Shayna Rosenbaum, Benjamin N. Cassidy, Katherine A. Herdman

**Affiliations:** ^1^Department of Psychology, York UniversityToronto, ON, Canada; ^2^Rotman Research Institute, BaycrestToronto, ON, Canada

**Keywords:** case study, developmental amnesia, hippocampus, extended hippocampal system, spatial learning, landmark recognition, mental navigation

## Abstract

The hippocampus is believed to have evolved to support allocentric spatial representations of environments as well as the details of personal episodes that occur within them, whereas other brain structures are believed to support complementary egocentric spatial representations. Studies of patients with adult-onset lesions lend support to these distinctions for newly encountered places but suggest that with time and/or experience, schematic aspects of environments can exist independent of the hippocampus. Less clear is the quality of spatial memories acquired in individuals with impaired episodic memory in the context of a hippocampal system that did not develop normally. Here we describe a detailed investigation of the integrity of spatial representations of environments navigated repeatedly over many years in the rare case of H.C., a person with congenital absence of the mammillary bodies and abnormal hippocampal and fornix development. H.C. and controls who had extensive experience navigating the residential and downtown areas known to H.C. were tested on mental navigation tasks that assess the identity, location, and spatial relations among landmarks, and the ability to represent routes. H.C. was able to represent distances and directions between familiar landmarks and provide accurate, though inefficient, route descriptions. However, difficulties producing detailed spatial features on maps and accurately ordering more than two landmarks that are in close proximity to one another along a route suggest a spatial representation that includes only coarse, schematic information that lacks coherence and that cannot be used flexibly. This pattern of performance is considered in the context of other areas of preservation and impairment exhibited by H.C. and suggests that the allocentric-egocentric dichotomy with respect to hippocampal and extended hippocampal system function may need to be reconsidered.

## Patterns of Preserved and Impaired Spatial Memory in a Case of Developmental Amnesia

Spatial memory of both newly encountered and well-known environments is crucial to everyday life—it enables us to navigate and to locate objects, buildings, and landmarks, and it may provide the necessary backdrop for the unfolding of episodic memories. Theoretical attempts to accommodate cross-species similarities in spatial navigation, and within-species specialization in episodic memory, have focused on the hippocampus, a brain region that is highly preserved across species, including humans. Cognitive Map Theory (CMT; O’Keefe and Nadel, 1978) and theories that derive from it (e.g., Byrne et al., 2007; Hassabis and Maguire, 2007) view the role of the hippocampus as dedicated to representing allocentric spatial relations among landmarks within environments. Egocentric (body-centerd) spatial representations, in contrast, are believed to be represented within regions outside of the hippocampus, including the posterior parietal cortex. However, the boundaries between what is allocentric and what is egocentric can be blurred, and the development of one coordinate system may or may not be orthogonal to the development of the other (see Baumann and Mattingley, 2014; Ekstrom et al., 2014; Wolbers and Wiener, 2014; this issue, for alternative interpretations). Studies show that the acquisition of allocentric spatial representations depends on hippocampal system integrity (e.g., Morris et al., 1982), but it is unclear whether with extended practice these representations come to rely on or can be acquired via brain structures within neocortex.

Spatial theories of hippocampal function have benefited from the study of individuals with early-onset hippocampal abnormalities who experience compromised episodic memory development and difficulties recollecting specific routes that may be necessary for everyday navigation (Vargha-Khadem et al., 1997). These areas of impairment occur in the context of at least some capacity for semantic knowledge acquisition that is slow, likely occurring over an extended period of time (Gardiner et al., 2008). Impaired episodic memory and spatial learning of newly encountered environments have been documented through the systematic investigation of the single developmental amnesic case Jon (Maguire et al., 2001; King et al., 2002) and in individuals aged 20–60 years who underwent unilateral temporal lobectomy for the relief of intractable epilepsy (Spiers et al., 2001). However, far less is known about the integrity of spatial representations of environments that have been navigated over many years. Here we investigate the possibility that spatial representations of extensively experienced environments may come to resemble semantic memories and withstand abnormal hippocampal development.

In a seminal study characterizing developmental amnesia in three cases (Vargha-Khadem et al., 1997), impaired spatial memory and wayfinding was featured as one of three primary deficits (Vargha-Khadem et al., 1997). The three cases were described as unable to “reliably find their way in familiar surroundings, remember where objects and belongings are usually located, or remember where they have placed them (p. 377),” a conclusion that was based on self report by caregivers and a single item on the Rivermead Behavioral Memory test requiring description of a route (Vargha-Khadem et al., 1997). More detailed examination of spatial memory involving the use of novel virtual reality environments in the well-studied case Jon confirmed difficulties in learning the location of objects (King et al., 2002, 2004) and in recognizing newly learned scenes presented from shifted viewpoints (Hartley et al., 2007). However, the integrity of spatial memory representations for familiar environments navigated extensively over many years has not been systematically investigated.

Additional insight into the role of the hippocampus has been gained from neuropsychological studies of adult-onset amnesic cases (Teng and Squire, 1999; Holdstock et al., 2000; Rosenbaum et al., 2000, 2005a; Corkin, 2002; Parslow et al., 2005). These individuals who, through injury or disease, sustained hippocampal damage in their adult lives, have highlighted the complex role that the hippocampus plays in spatial learning and memory. In line with predictions of CMT, research suggests that individuals with adult-onset amnesia are unable to form new, possibly allocentric, spatial representations of real-world environments (e.g., Teng and Squire, 1999; Holdstock et al., 2000; Rosenbaum et al., 2000; Nedelska et al., 2012) and virtual reality environments (e.g., Parslow et al., 2005; Goodrich-Hunsaker et al., 2010; see Ekstrom et al., 2003, for evidence of human hippocampal place cells), although there exists anecdotal evidence that H.M. was able to draw the layout of a house in which he had lived postmorbidly (Corkin, 2002).

While CMT is supported by studies on new spatial learning, findings from studies of remote spatial memory for environments learned long ago, prior to the onset of amnesia, might not have been predicted based on CMT. Indeed, anecdotal evidence in H.M. and other hippocampal amnesic cases of intact navigation in well-known environments suggested that at least some spatial relations contained within representations of well-known environments are less vulnerable to hippocampal damage compared to newly acquired spatial representations. These observations were followed up in systematic investigations of several amnesic cases. One such case, K.C., a person with large bilateral hippocampal lesions from a motorcycle accident (Rosenbaum et al., 2005b), was found able to make a variety of spatial judgments based on a large-scale, real-world environment (his home neighborhood) that he continuously experienced both before and after the onset of his amnesia (Rosenbaum et al., 2000). Some of these mental navigation tasks might have involved representing landmarks within an egocentric framework when sequencing landmarks along a route or describing a route from one familiar landmark to another. Other tasks appeared to require a more map-like representation of the spatial relations among landmarks, such as distance and direction, possibly within an allocentric reference frame (Rosenbaum et al., 2004; Ciaramelli et al., 2010), though it is possible that both coordinate systems were at play (Ekstrom et al., 2014).

K.C.’s performance on these tasks, which has been replicated in other adult-onset amnesic cases (e.g., patient E.P.: Teng and Squire, 1999; patient S.B.: Rosenbaum et al., 2005a; patient T.T.: Maguire et al., 2006; patient S.G.: Hepner et al., 2007), indicates preserved ability to retrieve remote spatial representations of very familiar environments that appear to be sufficient for actual navigation in those environments (Rosenbaum et al., 2000; for analogous findings in rodents, see Winocur et al., 2005, 2010a). This applies to tasks that were described by participants as solved primarily via a survey perspective, presumably within an allocentric framework, as well as those relying more on a ground-level perspective, presumably within an egocentric framework (Rosenbaum et al., 2004; Ciaramelli et al., 2010). Nevertheless, additional findings in cases K.C. and T.T. suggest that the patients no longer have available details contained within remotely learned environments that may be less essential to navigation, such as the appearance of houses and landmarks (Rosenbaum et al., 2000, 2005a) and non-major roads (Maguire et al., 2006).

Patterns of preserved and impaired aspects of remote spatial memory in hippocampal amnesia support the idea that the hippocampus is not needed for representing coarse or schematic spatial representations of large-scale environments that were formed long ago, similar to semantic memory. Conversely, fine-grained details of remotely learned environments continue to rely on hippocampal function, as in the case of episodic memory (Rosenbaum et al., 2001; Moscovitch et al., 2005). Separate but complementary evidence of slow semantic memory acquisition in developmental amnesia in the context of impaired episodic memory (Gardiner et al., 2008) offers support for the view that statistical regularities of multiple episodes or learning experiences can be extracted independent of the hippocampus (McClelland et al., 1995). Taken together, these findings raise the possibility that, in the absence of normal hippocampal development, semantic-like, schematic spatial representations of large-scale environments can nonetheless emerge following many years of active navigation within those environments, and that this learning may occur irrespective of the allocentric vs. egocentric nature of the task.

To investigate this possibility, we tested a well-studied developmental amnesic case, H.C., on her ability to represent two environments that she had frequented over an extended period of time: the neighborhood in which she has lived for the majority of her life and the downtown core of the city adjacent to her neighborhood. Anecdotally, H.C.’s ability to find her way around both newly encountered and well-known environments has been of great concern to her family over the years. H.C. and her family report that she relies on a Garmin GPS navigational system to navigate outside of her hometown but not within it. However, how she fares without such a device has not been systematically tested. As such, we tested H.C. on a range of mental navigation tasks that appear to place differential demands on allocentric vs. egocentric and schematic vs. detailed spatial representations (Rosenbaum et al., 2004, 2007; Ciaramelli et al., 2010). Comparisons were made with healthy controls who have similar experience with the two environments. These findings help determine the extent to which H.C.’s spatial memory for extensively navigated, real-world environments is similar to her significant impairment in forming episodic memories, as spatial theories of hippocampal function might predict, or similar to her ability to attain semantic knowledge over many years. What makes H.C. particularly well-suited to address these issues is that her amnesia is due to recently discovered congenital abnormalities that appear to have selectively targeted her extended hippocampal system, most strikingly the mammillary bodies and their key tracts (Olsen et al., 2013; Rosenbaum et al., 2014).

## Method

### Participants

**Figure 1:**
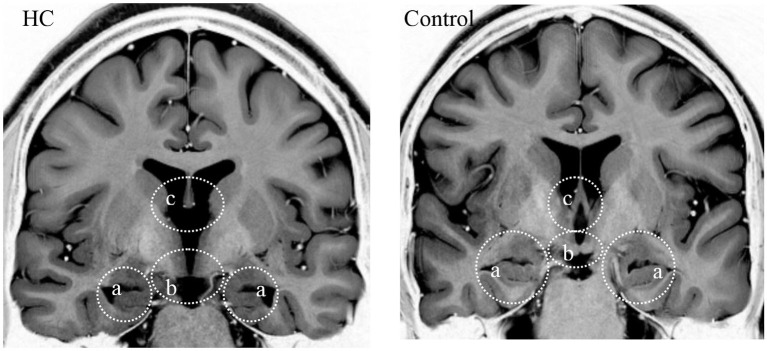
**Inverted coronal T2 images perpendicular to the hippocampus, showing poor digitation of the hippocampal head (a), and absence of the mammillary bodies (b) and anterior pillar of the fornix (c) in H.C. compared to an age-matched control**.

H.C. is a right-handed woman who was 22 years old at the time of testing. Manual tracing of high-resolution structural MR images indicates that, in comparison to demographically matched controls, H.C.’s hippocampus is reduced in volume by 30% bilaterally, and this reduction is generally consistent across subfields (Olsen et al., 2013). The anterior thalamic nuclei are similarly reduced (Rosenbaum et al., 2014), whereas other medial temporal lobe structures are normal in volume (Olsen et al., 2013). Additional careful analysis of her extended hippocampal system indicates agenesis of the mammillary bodies, abnormal hippocampal shape and orientation, and rerouting of her fornices, pointing to a congenital basis for her memory impairment (Figure 1; Rosenbaum et al., 2014). A separate study investigating H.C.’s declarative memory reported episodic memory impairment on tests of personal and public event memory, but intact personal and general semantic memory (Rosenbaum et al., 2011), a general dissociation that has been reported in other amnesic cases with hippocampal system pathology (e.g., Gadian et al., 2000; Rosenbaum et al., 2005b, 2008; Gilboa et al., 2006; Vicari et al., 2007; Bindschaedler et al., 2011; Picard et al., 2013; earlier cases reviewed in Moscovitch et al., 2006; but see Bright et al., 2006; Kirwan et al., 2008). Significant impairments in both recollection and familiarity were also reported in relation to H.C.’s performance on several lab-based tests of recognition memory (Rosenbaum et al., 2011). Another study demonstrated that her deficits in past remembering also extended to her ability to imagine the future (Kwan et al., 2010; but see Hurley et al., 2011) and to imagine the experiences of familiar others (Rabin et al., 2013). Despite these areas of difficulty, H.C. successfully graduated from high school and attended community college for 2 years, totaling 14 years of formal education. She also successfully obtained a driver’s license at the age of 17 and, at the time of testing, had 6 years of driving experience around her neighborhood and the core part of her city. She participated in a rehabilitative program focused on systematic procedural training and has learned to use various handheld electronic devices and organizing software to assist her with scheduling daily activities, getting directions and keeping track of newly encountered individuals. Through this training, she has gained a greater sense of autonomy in her day-to-day life. A detailed neuropsychological examination has confirmed that she has average intelligence and does not demonstrate any further cognitive difficulties beyond her memory impairments (Rosenbaum et al., 2011; see Table 1 for a summary of relevant findings from neuropsychological testing).

**Table 1:** **Neuropsychological profile of H.C**.

Test	Raw score	Normed score
**Intellectual function and academic attainment**
WASI		Percentile
Verbal IQ	104	61
Performance IQ	106	66
Full scale IQ	106	66
AM-NART		Standard score
Total correct	27	101.28 (estimated FSIQ)
WAIS-III		Scaled score
Arithmetic	10	8
Information	19	12
**Language**		Percentile
Boston naming test^1^	58	77–79
Semantic fluency (animals)	32	>90
Phonemic fluency (FAS)^2^	53	70–80
WASI		*T*-score
Vocabulary	58	55
**Anterograde memory**		
WMS-III		Scaled score
Logical memory I	27	4
Logical memory II	3	1
California Verbal Learning Test-II		*z*-score
Total trials 1–5	44	38 (*T*-score)
Short delay free recall	0	−4
Short delay cued recall	5	−3.5
Long delay free recall	3	−3
Long delay cued recall	4	−3.5
Recognition	13	−2
Rey Osterreith complex figure^3^		*T*-score
Immediate recall	4	<20
Delayed recall	3	<20
Delayed recognition	17	22
**Processing Speed**		
WAIS-III		Scaled score
Digit Symbol	96	13
Symbol Search	45	14
**Visuospatial Function**		Percentile
Judgment of line orientation	24	56
Benton facial recognition	45	33–59
Rey-osterrieth complex figure—copy^3^	33	>16
WASI		*T*-score
Block design	52	54
**Attention and executive function**		
Stroop^4^		*z*-score
Word full (sec)	45	3.65
Color full (sec)	48	−0.03
Interference full (sec)	80	−0.57
Trail making test^1^		*z*-score
Part A (sec)	34	0.69
Part B (sec)	55	−0.23
WASI		*T*-score
Similarities	35	50
Matrix reasoning	29	55
WAIS-III		Scaled score
Digit span forward	10
Digit span backward	5
Digit span total	15	8
Wisconsin card sorting task		*T*-score
Categories^5^	10	57
Perseverative errors	10

*Note. AM-NART, American National Adult Reading Test; WASI, Wechsler Abbreviated Scale of Intelligence; WAIS-III, Wechsler Adult Intelligence Scale–III. Additional results of neuropsychological testing reported in Rosenbaum et al. (2011), Hurley et al. (2011), and Rabin et al. (2012). ^1^Spreen and Strauss (1998), ^2^Tombaugh et al. (1999), ^3^Meyers and Meyers (1996), ^4^in-house unpublished normative data, ^5^Heaton et al. (1993)*.

H.C.’s performance was compared to that of 7 controls (5 female, 2 male)^1^ matched for age (mean = 26.5, SD = 12.34) and education (mean = 14, SD = 1.79), all of whom continue to live in H.C.’s neighborhood, other than 1 control who moved away and has not visited in the 4 years prior to testing. Six participants were recruited through word of mouth and one participant was recruited through an advertisement. One female participant was excluded from the analyses due to a previous diagnosis of ADEM. All participants provided informed consent and received monetary compensation for participating in accordance with the research ethics boards of both York University and Baycrest.

### Experimental Measures and Procedure

Nine mental navigation tasks were adapted from previous studies (Rosenbaum et al., 2000, 2004, 2005a, 2007, 2012; Ciaramelli et al., 2010) to test participants’ spatial memory for their home neighborhood and for the adjacent downtown city core. Both represent spatial environments frequently navigated by HC and controls and are approximately equal in spatial area (~6 km^2^). Tasks were constructed to assess memory for the visual identity, the locations, and the distance, direction, and routes between a set of 37 well-known landmarks dispersed throughout both environments. Based on previous findings, some tasks appear to rely to a greater extent on an allocentric framework and others on an egocentric framework, as detailed in Table 2 (see also Rosenbaum et al., 2004; Ciaramelli et al., 2010). Tasks were completed by all participants in a fixed order as listed below, as the completion of some tasks could influence performance on other tasks if administered in a different order. For instance, on the sketch mapping test, it was important for participants to recall landmarks without the aid of landmark or street names, which are provided in other mental navigation tasks. Instructions for each task were made available for the duration of the task for reference.

**Table 2:** **Performance on the mental navigation tasks**.

Spatial task	Description	Dominant reference frame^1^	Type of score	H.C.’s score	Healthy controls’ mean score (SD)
Sketch mapping	Draw a detailed map of the streets and landmarks located in well-known neighborhood and downtown environments	A	a) Total landmarks b) Total street segments c) Total details (landmarks + segments)	a) 20** b) 34 c) 54**	a) 44.833 (8.660) b) 106.167 (35.254) c) 151 (35.254)
Landmark localization	Locate landmarks on an outline map	A	a) Mean distance error (km) for neighborhood landmarks b) Mean distance error (km) for *city core* landmarks c) Total mean distance error (km)	a) 0.37* b) 0.762*** c) 0.566***	a) 0.247 (0.072) b) 0.273 (0.118) c) 0.26 (0.086)
Proximity judgments	Of two landmarks, select the one that is closest to a third, reference landmark	A	Accuracy (Proportion correct)	0.7	0.817 (0.075)
Distance judgments	Provide distances between pairs of landmarks	A	Mean distance error (km)	0.7	1.806 (2.117)
Landmark sequencing	Place a series of 15 landmarks in the correct order from north to south	E	Accuracy (Proportion in correct sequence)	0.8*	0.933 (0.073)
Vector mapping	Draw a line indicating distance and direction from a specified landmark to a second named landmark	A	a) Mean distance error (km) b) Mean angle error (degrees)	a) 0.197 b) 13.5	a) 0.239 (0.076) b) 13.638 (6.089)
Blocked route^2^	Describe most efficient detour between 2 landmarks, given that the most direct route is blocked	A/E	a) Proportion correct (# correct/total) b) Proportion correct, outlier removed	a) 0.6 b) 0.6**	a) 0.763 (0.204) b) 0.84 (0.082)
Landmark recognition	Of two landmarks, select the one that is located in the neighborhood or downtown environment	NA	Accuracy (Hit rate—false alarm rate)	0.973	0.95 (0.032)

Note. A, allocentric; E, egocentric

*^1^As reported in Ciaramelli et al. (2010) and Rosenbaum et al. (2004, 2007)*.

*^2^One outlier was identified in the control sample and included in analysis a) but removed from analysis b)*.

***Significant difference at *p* < 0.01; **Significant difference at *p* < 0.05; *Approaching significance (*p* < 0.1).

#### Sketch Mapping

Participants were given an 8.5 × 11 inch sheet of white paper marked with both metric and imperial scales and were asked to draw as accurately as possible a detailed map of their home neighborhood, including as many of the streets and landmarks as possible. They were asked to pay attention to scale and were given the names of streets that bounded the north, west, south, and east of the neighborhood. Participants were given up to 20 min to complete the task. This process was repeated for the downtown city environment.

Using a modified version of Blades (1990) sketch map information content, the number of landmarks (easily identifiable and/or functionally relevant static structures representing nodes) and the number of street segments (named or unnamed segments of streets, roads, or transit passages that must be attached on at least one end to a landmark or neighboring street segment) were tabulated by a blind rater for home neighborhood and downtown core sketch maps for each participant. Overall configuration of the maps was noted.

#### Landmark Localization

Participants were again given an 8.5 × 11 inch sheet of white paper marked with both metric and imperial scales, with the streets bounding the north, west, south, and east of the neighborhood demarcated. Participants were given the names of 10 landmarks and were asked to mark as accurately as possible the locations of each landmark on the paper using the scale and the major streets as guidelines. This process was repeated for the downtown city environment. Participants’ performance on this task was assessed by calculating the mean landmark localization error as measured by the linear distance (km) between the correct placement of each of the landmarks and the participant’s responses.

#### Proximity Judgments

In each of 10 trials, participants were presented with 3 photographs of well-known landmarks from the two environments, presented in a triangular configuration (a reference landmark at the top and two choice landmarks at the bottom) on a laptop computer screen with E-prime software (Psychology Software Tools, Pittsburgh, PA). Participants were instructed to decide which of the two choice landmarks is closest in distance to the reference landmarks with a button press (left corresponding to the bottom left landmark and right to the bottom right landmark). The level of difficulty was manipulated by including nearly equivalent distances between each of the two choice landmarks and the reference landmark in some trials and by incorporating landmarks from both environments within the same trial. Accuracy (proportion correct) was calculated.

#### Distance Judgments

Participants viewed 10 pairs of photographs, each depicting a well-known landmark from one of the spatial environments and asked to give their best estimate of the absolute distance between the two in their preferred unit of measurement (metric or imperial). All of the participants’ responses were converted to km, and absolute distance error was calculated by subtracting the actual distance from the estimated distance. The error scores for each trial were then averaged, resulting in an average error score for each participant.

#### Landmark Sequencing

Fifteen randomly ordered photographs of well-known landmarks from both environments, located along a north-south route, were combined and presented to participants. Participants were asked to imagine traveling from north to south and to sequence the landmarks in the order that they would be passed along the route. The total number of landmarks that were correctly sequenced with respect to an adjacent landmark was calculated for each participant and divided by 15, providing a Landmark Sequencing accuracy score.

#### Vector Mapping

Each participant was given a set of 10 sheets of 8.5 × 11 inch paper, 5 depicting an outline map of the neighborhood and the other 5 depicting a map of downtown. In addition to the 4 boundary streets corresponding to one of the two environments, participants were provided with a dot indicating the actual location of a landmark and asked to draw a line depicting as accurately as possible the distance and direction from the given landmark to a second named landmark. Deviation of the line drawn between each pair of landmarks from the actual line was calculated separately for the length (distance) and angle (direction) of the line and averaged across trials.

#### Blocked Routes

In each of 5 trials, participants were given the names of two landmarks, the first representing the start location and the second the goal location, from one or both of the environments. A name of a major street along the most direct route between the two landmarks was also provided and participants were informed that this particular street would be inaccessible for the trial—the street could be crossed but not taken. Participants were then asked to imagine traveling from the start landmark to the goal landmark, avoiding the inaccessible street while adhering to the rules of the road (e.g., respecting one-way streets), and to describe the most direct detour to reach the goal. The correct route for each trial consisted of 4 key left and/or right turns. Proportion of correct turns across all 5 trials was calculated for each participant from a raw score out of a possible 20.

#### Landmark Recognition

This task does not measure the ability to represent spatial relations between landmarks, but rather measures the “what” of spatial memory—the participant’s ability to visually represent environmental features and navigational cues within the spatial environments. It also ensures that the participants did not have trouble identifying the well-known landmarks chosen for the study, which could have impeded their performance on the mental navigation tasks. Participants were presented with a randomly ordered set of 37 well-known landmarks from both environments and 37 closely matched, unfamiliar distractor landmarks gathered from other Canadian towns and cities that were similar in terms of age of building, architectural style, and function. For each landmark, participants were asked whether it is located in either of the familiar environments, and if so, to identify it. An accuracy score, calculated by subtracting the false alarm rate from the hit rate, was used in the statistical analysis for comparison of performance.

## Results

Statistical analyses involved the use of a modified *t*-test procedure established by Crawford and Howell (1998) for comparing a single case to a small control sample. All of the mental navigation tasks along with the participants’ corresponding results are summarized in Table 2.

### Mental Navigation Tasks

**Figure 2:**
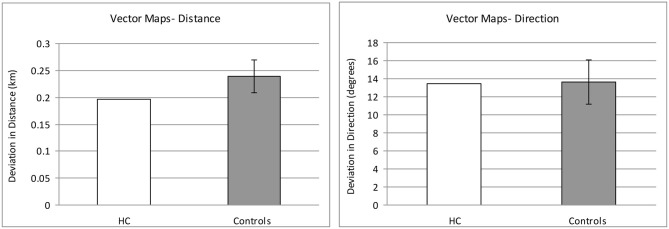
**Mental navigation performance in H.C. and controls on the vector mapping test, as measured by deviation in distance (left) and direction (right)**. Error bars indicate ± one standard error.

**Figure 3:**
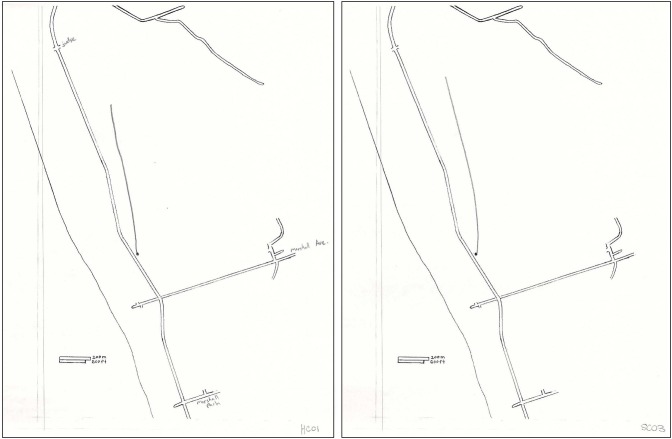
**Sample of a vector mapping trial**. A vector representing the estimated distance and direction between two landmarks was drawn by H.C. (left) and a control (right) on an outline map of a familiar downtown city environment, with one of the landmarks specified by a dot on the map. The actual size of the environment is approximately 6 km^2^.

Remarkably, despite a hippocampus and extended hippocampal system that did not develop normally, H.C. demonstrated intact performance compared to controls on several of the mental navigation tasks, namely proximity judgments (*t* = −1.444, *p* = 0.104), distance judgments (*t* = −0.483, *p* = 0.325), and vector mapping (direction: *t* = −0.021, *p* = 0.492; distance: *t* = −0.512, *p* = 0.315; the data, along with a representative vector drawn by H.C. and a control, are presented in Figures 2, 3, respectively). In contrast, H.C. showed a trend towards significant impairment on the landmark sequencing task (*t* = −1.691, *p* = 0.076), and her performance was significantly impaired on the landmark localization task (*t* = 3.294, *p* = 0.011).

An outlier was identified in the data for the blocked route task. Control participant 004 performed much worse than other controls and H.C. only on this task. The reason for his poor performance is unknown, which is why we present the results with and without his data point. When the outlying data point is included in the analysis, the results suggest that H.C. did not differ significantly from controls (*t* = −0.740, *p* = 0.246). However, when the outlier is withheld from the analysis, H.C. appears to perform significantly worse than controls (*t* = −2.672, *p* = 0.028). It is important to note that responses were strictly scored based on the use of the most direct detour between two landmarks, for which H.C. demonstrated a deficit. However, this method of scoring did not take into account participants’ general ability to navigate between two landmarks using a detour, regardless of its length. For example, H.C. received a raw score of 0 (out of a possible 4) on one of the five routes tested, as she did not use the most direct route. She did, however, correctly navigate to the goal landmark, albeit along a longer route than necessary (distance along H.C.’s route: 2.9 km; distance along most direct route: 1.9 km). Thus, H.C.’s overall route descriptions were feasible but limited in terms of flexible use of existing spatial representations.

**Figure 4:**
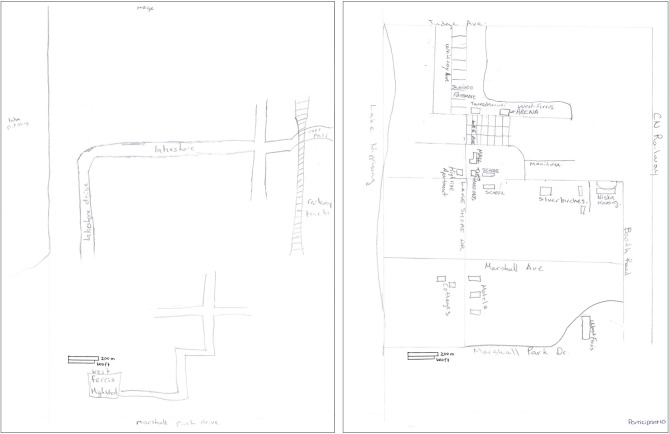
**Sketch map of H.C.’s home neighborhood as drawn by H.C. (left) and a control (right)**. The actual size of the environment is approximately 6 km^2^.

**Figure 5:**
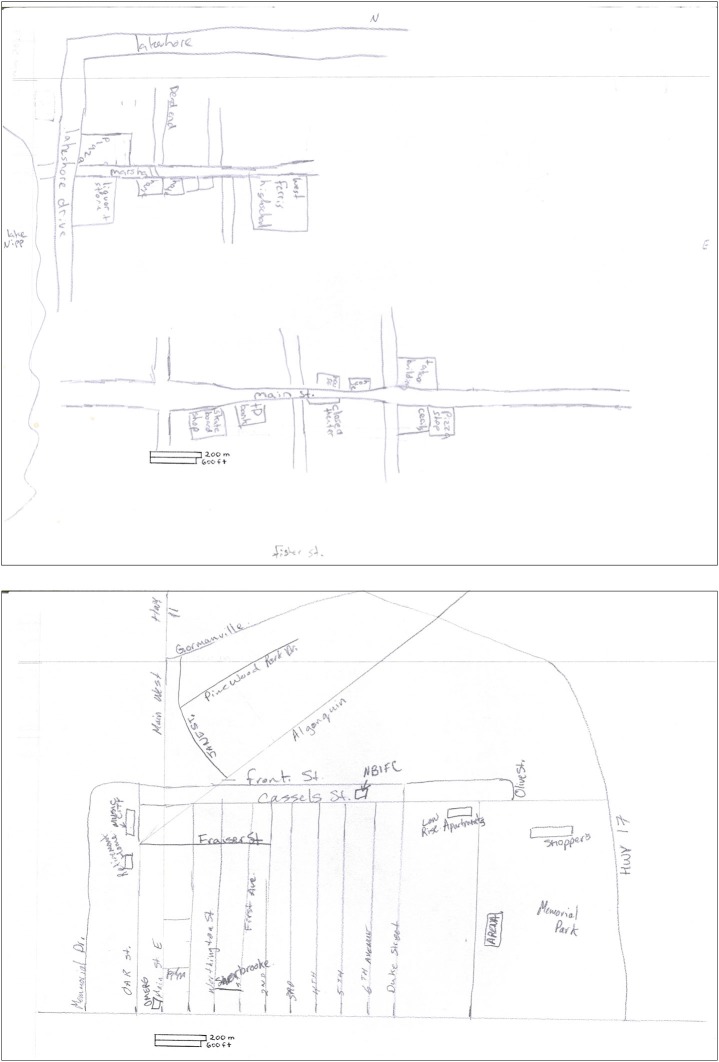
**Sketch map of a downtown city environment adjacent to H.C.’s neighborhood as drawn by H.C. (top) and a control (bottom)**. The actual size of the environment is approximately 6 km^2^.

On the sketch mapping task, H.C. included roughly one third the number of total details (landmarks and street segments) as did controls, which represents a significant difference (see Table 2). Qualitatively, all participants showed some difficulty scaling the landmarks, streets, and topographical elements appropriately in their sketch maps, even though their attention was directed to the scale that was provided. Aside from this consistent misrepresentation of the two spatial environments in all participants’ sketch maps, H.C.’s two sketch maps appeared to be considerably impoverished when compared to the maps of controls (see Figures 4, 5). H.C.’s sketch map of the neighborhood was mostly limited to landmarks and streets located along the demarcated border of the neighborhood. The lake that was described to her as located on the left of the paper (corresponding to the western border of the neighborhood) was drawn as such by H.C., but it did not extend the full length of the page as it should have. The intersection that was identified as bordering the top of the paper (corresponding to the northern border) was included but placed inaccurately (on the right of the paper instead of centered at the top). The railroad tracks, which frame the eastern and northeastern borders were drawn only on a portion of the right (east) side. This may account for the incorrect placement of the intersection, as the tracks cross one of the streets of the intersection, but only as they curve northwest across the region represented on the sketch map. H.C. included the name of the street that was described as bordering the bottom of the paper (corresponding to the southern border). When traveling east on this street, one would arrive at her high school, but H.C. placed her high school in the bottom left corner of the paper, along with a few unidentified streets connected to it. Some of the streets that she drew could be interpreted as a portion of the streets that fall along a route she has taken to her high school, but she neglected to label these streets, provide any continuation of them beyond the portions that are relevant to the route, or draw any of the side streets that branch off of the streets depicted. The main street that runs northwest to southeast through the extent of the neighborhood, along which many of the landmarks from the other mental navigation tasks are located, was drawn by H.C. in the center of the page and possesses a sharp bend that does not appear to exist on an actual map. It is important to note that the house in which H.C. has lived since birth was not placed on this map, nor were any of the other landmarks (aside from her high school) to which she navigated on a daily basis since childhood.

H.C.’s sketch map of downtown is similarly degraded compared to that of controls (see Figure 2) and even lacked two of the four features/streets that were provided in the written instructions as boundaries of the environment. Only the lake that extends along the left side of the paper (west end of the map) and the name of the street at the bottom of the paper (south end of the map) were included. A portion of the main street was included with 8 landmarks accurately placed along its length, but the street was drawn perpendicular to how it appears on an actual map. Interestingly, H.C. inserted a portion of her home neighborhood, which includes her high school and two other landmarks, in the top left (northwest) corner of her downtown sketch map, even though these features belong along the southern border of her neighborhood sketch map.

Finally, as indicated in Table 2, H.C.’s recognition and identification of landmarks did not differ significantly from the performance of controls. This helps verify that any findings of impaired performance on the mental navigation tasks are unlikely to be due to an inability to recognize the landmarks themselves.

## Discussion

We report a detailed investigation of the capacity to represent both coarse and fine-grained spatial information acquired over an extended period of time in the face of congenital abnormalities of the hippocampus and extended hippocampal system in the well-characterized case H.C. H.C. was tested on a wide range of tasks designed to measure her memory for the spatial relations and visual identity of landmarks contained within her home neighborhood and downtown city core that she has navigated for many years. Consistent with evidence of semantic learning in H.C. and in other developmental amnesic cases, H.C.’s performance on tests of distance and direction indicate that she has acquired spatial relational information on tests that are typically achieved from a survey perspective within allocentric coordinates. Some of these judgments required integration of spatial information across separately demarcated environments that are not directly adjacent to one another. H.C.’s ability to describe routes between familiar locations is generally intact, but her ability to devise the most efficient route appears to be compromised. Moreover, though she appeared to have acquired the overall layout or spatial “gist” of well-known environments, memories for those environments were significantly lacking in detail. This pattern of preserved and impaired performance resembles in some respects H.C.’s impaired episodic memory but intact semantic learning and appears to defy clear distinctions between allocentric and egocentric representations.

The current results provide evidence that the visual identity of landmarks, and the spatial relations among them, can be acquired without a structurally intact hippocampus and extended hippocampal system. Indeed, H.C. correctly identified landmarks located in familiar neighborhoods from photos and distinguished those landmarks from similar-looking distractor landmarks. She was also able to retrieve the relative and absolute distances between pairs of known landmarks on proximity and distance judgment tasks, respectively, and provide accurate estimates of both distance and direction on a vector mapping task. These findings are consistent with reports in adult-onset amnesic patients of intact spatial memories of remotely learned environments, at least those that are schematic in nature, despite extensive hippocampal damage and impaired acquisition of newly encountered environments (Teng and Squire, 1999; Rosenbaum et al., 2000, 2005a,b; Maguire et al., 2006). It is important to note that tasks of spatial memory are rarely (if ever) process-pure, and are likely to engage a blend of egocentric and allocentric processes to varying degrees (Ekstrom et al., 2014). Thus, H.C. might have used an egocentric or stimulus-response strategy to achieve intact performance (Bohbot et al., 2007; Etchamendy et al., 2012; Bohbot, 2015). Although this explanation might apply to H.C.’s performance on the proximity and distance judgment tasks, vector mapping requires participants to disregard specific routes between landmarks and is more likely to induce a survey perspective. Intact vector mapping performance in H.C. appears to be inconsistent with the CMT view that the hippocampus is necessary for the acquisition, maintenance, and retrieval of allocentric spatial memories (O’Keefe and Nadel, 1978; Burgess, 2008).

In contrast, H.C.’s seemingly poor performance on the blocked route task is perhaps less surprising in the context of findings in previously studied developmental amnesic cases, which had been taken as support for CMT (Vargha-Khadem et al., 1997). However, the area of impairment in these cases was in describing well-known routes. H.C., in contrast, is able to provide accurate descriptions of routes that were known to her from many years of experience navigating them, but the routes that she devises are not the most direct between locations when faced with a road block. The blocked route task, viewed as a paradigmatic test of hippocampal function (O’Keefe and Nadel, 1978; cf. Tolman, 1948), requires the flexible use of existing representations for successful navigation. A previous challenge in creating a blocked route task based on highly familiar, contained neighborhood environments is that participants likely experienced the many possible combinations of routes between landmarks, such that detour routes were deemed as familiar as the most direct routes (Rosenbaum et al., 2004, 2007). Whereas the control participants may have approached the task the way that they would a direct route task that does not involve navigating around a barrier, H.C.’s less efficient learning suggests that she likely treated the secondary routes as novel. Importantly, H.C.’s difficulties performing the blocked route task appear to play out in everyday life: H.C. has been described by family members as capable of navigating in familiar places unless her attention is diverted and she is required to recalibrate her route from her new start location. Nevertheless, that she is able eventually to describe acceptable routes that are not random but purposeful, consistently leading to the goal destination, indicates that her long-standing spatial representation, though perhaps less flexible than that of controls, may be allocentric in nature but based on a coarse or schematic representation.

Dissociations observed in H.C.’s sketch maps provide further evidence that her “cognitive map” contains only the very coarse, schematic elements of the two familiar environments on which she was tested. H.C.’s acquisition of more fine-grained, detailed features of the environments did not seem to benefit from extended navigational experience, as indicated by her sketch maps, which were impoverished in terms of the number of streets and landmarks that she had included. The overall configuration seems to have been disrupted by the absence of some details and inaccurate placement of others. Another possibility is that her impoverished sketch maps reflect a retrieval deficit, whereby detailed spatial information is not automatically conjured up in memory and requires more intensive cueing. However, even when given the names of landmarks to localize in the landmark localization task or to include as boundaries on the sketch maps, she placed them inaccurately or failed to include them, suggesting that it is unlikely that her sketch map performance was due to impaired retrieval.

We have reported comparable dissociations in adult humans and rats with hippocampal lesions who show extensive, ungraded loss of remote memory acquired long before lesion onset if the memory continues to rely on its initial, detailed context, but sparing of remote memory if the memory is transformed from a contextually dependent one to one that is more semantic or schematic (Rosenbaum et al., 2001; Moscovitch et al., 2005, 2006; Winocur et al., 2005, 2010a, b). Here, we extend this distinction to show that new spatial learning is possible when it occurs over many years, but the representations are not sufficient to support the precise placement of multiple landmarks in relation to each other when they are located in close proximity, unlike findings in adult-onset amnesic cases. Differences between H.C.’s performance and that of adult-onset amnesic cases may reflect the involvement of different processes when spatial learning takes place before vs. after loss of hippocampal integrity. Research in rats with selective hippocampal lesions that received extensive post-operative rearing in a “village maze,” a complex, enriched environment with multiple incentives to explore, designed to simulate real-world navigation conditions in humans, might be particularly informative (Winocur et al., 2010a). Hippocampally lesioned rats reared over a 3-month period post-operatively showed evidence of new spatial learning, but this reflected benefits from directed training at test and not savings from extensive rearing prior to test. By contrast, lesioned rats reared pre-operatively did show significant savings from their rearing experience (Winocur et al., 2005), even after only 2 weeks of pre-operative rearing (Winocur et al., 2010a). Importantly, in both groups, a series of probe trials showed that the rats’ performance was, indeed, based on the application of allocentric spatial strategies and not on the use of non-spatial local cues or procedural learning.

Though the hippocampus has long been considered essential for allocentric spatial memory and navigation, the current findings in H.C., together with findings in adult-onset amnesic cases and hippocampally lesioned rats, support the view that the hippocampus may be needed perpetually for the acquisition and/or long-term maintenance of spatial-perceptual details of those representations that allow the individual to re-experience an environment through which he or she is navigating. By contrast, coarse spatial memories that are sufficient for navigation can be acquired and maintained by extra-hippocampal structures such as the parahippocampal, retrosplenial, and posterior parietal cortices (e.g., Bohbot et al., 1998; Rosenbaum et al., 2004, 2007; Bohbot and Corkin, 2007; Ciaramelli et al., 2010; Hirshhorn et al., 2012a,b; Epstein and Vass, 2013; Baumann and Mattingley, 2014). In non-amnesic individuals, the memories are transformed over time from detailed, perceptual representations (scenes) that allow one to richly re-experience the environment to more schematic representations or “maps” that consist of well-known landmarks and the approximate relations between them, and that are sufficient for navigation. As memories are transformed from scenes to schematic maps, they lose their dependence on the hippocampus. The two types of memory, however, can co-exist and interact with each other as tasks demand. Findings in H.C., together with previous evidence of new spatial learning in H.M. and in rodents with hippocampal lesions trained and tested post-operatively further suggest that, like semantic memories, spatial memories can be formed independently of the hippocampus. One possibility is that they emerge from the gradual abstraction in the neocortex of statistical regularities in the environment, and do not depend on hippocampal system integrity for their acquisition and maintenance (McClelland et al., 1995; Kumaran and McClelland, 2012). Neocortical structures function to extract commonalities among landmarks and routes that have been approached from multiple angles and perspectives in the context of different goal states and motivating factors under naturalistic conditions. This representation provides a skeletal architecture into which event and spatial details may be incorporated. Without hippocampal input, however, the familiar environment is represented as a collection of disjointed spatial features (e.g., landmarks), each of which can be referenced on its own, but that is not linked with other features in a configural way (Winocur et al., 2010a,b).

The current findings are compatible with other influential theories of hippocampal function. The areas of impairment described in the current study may be interpreted in terms of a core deficit in relational memory (Eichenbaum and Cohen, 2001; Moses and Ryan, 2006; Watson et al., 2013; see also Rose et al., 2012; Olsen et al., 2015 for a relational memory explanation of other areas of impairment exhibited by H.C.). By this view, H.C. is unable to represent and integrate multiple elements, and spatial relations among elements, into a flexible, coherent representation of large-scale space. This could explain her preserved ability on tests of distance and direction between pairs of landmarks but difficulties assembling spatial relations among many landmarks on the sketch map, localization, and sequencing tasks that require the online processing of individual landmarks in relation to multiple surrounding landmarks. This is best illustrated by H.C.’s performance on the landmark sequencing task, which approached significant impairment. Closer inspection of her responses reveals that she placed 12 of the 15 landmarks adjacent to at least one of the correct neighboring landmarks and in the correct order, and an additional 2 landmarks adjacent to a neighboring landmark but in the incorrect order (i.e., swap errors; see Watson et al., 2013 for a similar finding in amnesia in relation to reconstructed locations of studied objects within an array). The three landmarks that were out of sequence were situated in the middle of the given sequence, with the actual distance between them minimal. These landmarks might occupy an overlapping representation and may be further interpreted as a failure in pattern separation (Yassa and Stark, 2011; Copara et al., 2014; Kesner and Rolls, 2014; see also Marr, 1971; McClelland et al., 1995). H.C.’s pattern of preserved and impaired performance does not fit neatly within a simple allocentric-egocentric dichotomy but may be compatible with CMT inasmuch as the hippocampus and structures that are intimately connected to it are viewed as necessary for the flexible implementation of allocentric representations.

Finally, we show that relatively selective disruption of the extended hippocampal system (fornix, mammillary bodies, and anterior nucleus of the thalamus), with abnormally rotated hippocampi that are reduced in volume by a minimal, yet significant amount (Olsen et al., 2013; Rosenbaum et al., 2014), is enough to impede rapid place learning. Instead, such damage can yield more schematic and less coherent spatial memories that consist of rudimentary links among elements that are not fully integrated with one another and that cannot be used in novel ways when confronted with a road block (for similar conclusions in hippocampally lesioned rodents, see Winocur et al., 2010a; see also Hassabis et al. (2007)). Though laboriously formed, possibly over many years, these representations are sufficient to support real-world navigation, as suggested by anecdotal evidence that H.C. is capable of navigating in well-known environments under certain conditions. It remains possible that the seemingly limited nature of structural abnormalities within H.C.’s hippocampus that occurred in early fetal development allows for at least some functional capacity that is sufficient to support those aspects of spatial memory that appear to be intact in H.C. We view this as unlikely given the classic pattern of impaired episodic memory in the context of otherwise intact intellectual and cognitive function on standard neuropsychological tests, along with significant abnormalities within the major output of the hippocampus. Nevertheless, fMRI could be used to reveal the functional integrity of H.C.’s hippocampal tissue and/or neocortical regions supporting H.C.’s intact performance, an approach taken in studies of remote spatial memory in K.C. (Rosenbaum et al., 2007) and scene construction in P01 (Mullally et al., 2012) and the developmental amnesic case Jon (Mullally et al., 2014).

To conclude, a person with congenital hippocampal system pathology and impaired episodic memory has managed to form spatial memories of large-scale, real-world environments. Despite an intact ability to represent distance and direction between pairs of landmarks, H.C.’s difficulties devising the most direct route between landmarks, producing detailed spatial features on maps, and accurately ordering more than two landmarks that are in close proximity to one another along a route are interpreted as symptomatic of a spatial representation that includes only coarse, schematic information that lacks coherence and that cannot be used flexibly. The current study adds to the literature by confirming early findings based on cursory measures of impaired spatial memory in developmental amnesia, and by explaining the possible basis of the impairment in greater detail while showcasing its limits. The original conclusion of impaired wayfinding in familiar, real-world environments in the seminal paper on patient Jon and two other developmental amnesic cases (Vargha-Khadem et al., 1997) is limited to the patients’ route descriptions when they were still very young. It is possible that developmental maturation, along with intensive experience navigating within home and downtown environments over many years, may have been responsible for areas of intact performance in H.C. Future research that tracks the course of new spatial learning from initial encoding, analogous to the slow semantic learning evident in patient Jon (Gardiner et al., 2008), would be of great value in determining the extent to which frequent navigation over a long period of time from multiple perspectives is necessary conditions for the development of such maps.

## Conflict of Interest Statement

The authors declare that the research was conducted in the absence of any commercial or financial relationships that could be construed as a potential conflict of interest.

## Footnotes

^1^Although there is some evidence of sex differences in the spatial learning literature, there is no clear evidence of systematic sex-related differences in performance in the current study or, to our knowledge, in previous studies of remote spatial memory that employ similar tests to the ones used in the current study.
